# Death of an Ulster Practitioner

**Published:** 1911-09-30

**Authors:** 


### Death of an Ulster Practitioner.

The death is announced of Dr. William Twigg, of Dungannon, Tyrone. He had been in practice in Dungannon for half a century, short of a few months, and had always taken a great interest in local parish affairs. He qualified in 1861 and took the M.D. degree of the Queen's University of Ireland in the following year. Since 1864 he held the appointment of Medical Officer of the Union Workhouse and the Fever Hospital. Dr. Twigg also belonged to the Mid-Ulster Artillery for many years, retiring only in 1901 with the rank of Surgeon-Lieutenant-Colonel. His funeral on September 13 was largely attended.

